# Response to “Historical Circulation and Forgotten Evidence of Oropouche Virus in Colombia: Not as New as it Seems”

**DOI:** 10.4269/ajtmh.25-0423b

**Published:** 2025-09-11

**Authors:** Rebecca C. Christofferson, Berlin Londono-Renteria, Christopher N. Mores

**Affiliations:** Louisiana State UniversitySchool of Veterinary MedicineBaton Rouge, Louisiana, USAE-mail: rcarri1@lsu.edu; Department of Tropical Medicine and Infectious Disease, Tulane UniversityNew Orleans, USAE-mail: blondono@tulane.edu; George Washington UniversityWashington D.C., USAE-mail: cmores@gwu.edu

 Authors Silva-Ramon and Faccini-Martínez, in their letter to the Editor, “Oropouche Virus in Colombia, Not as New as it Seems” are absolutely correct that historical context is necessary to fully understand emergent arboviruses such as Oropouche virus (OROV). This was indeed a primary motivation of our study. We appreciate the additional references describing serologic studies of OROV exposures in Colombia identified in their letter. In our study, we took the specific and unambiguous approach to look for confirmed Oropouche cases (those with viral RNA still detectable in serum) among febrile patients.^1^ While ours remains at the time, the first detection of active OROV infection this westerly in Colombia, the overall conclusion that OROV circulation in Ocana represented the most westerly geography is inaccurate. However, it is important to note that the presence of antibodies is less correlated in time and space than active viremia. It was not our intention to conflate total exposure history in Colombia with detection in the acute phase of disease. We thank Silva-Ramon and Faccini-Martínez for bringing to our attention these additional sources of data and are happy to have a more complete picture of OROV in Colombia.
